# Generalization of visuomotor adaptation depends on the spatial characteristic of visual workspace

**DOI:** 10.1007/s00221-012-3264-8

**Published:** 2012-09-19

**Authors:** Lei Wang, Jochen Müsseler

**Affiliations:** Department of Work and Cognitive Psychology, RWTH Aachen University, Jägerstraße 17-19, 52066 Aachen, Germany

**Keywords:** Distal action space, Visuomotor adaptation, Sensumotor adaptation, Generalization, Action control

## Abstract

The present study aims to address a novel aspect of visuomotor adaptation and its generalization. It is based on the assumption that the spatial structure of the distal action space is crucial for generalization. In the experiments, the distal action spaces could manifest either a symmetric or parallel structure. The imposed visuomotor rotations in the adaptation and the following generalization were either the same or opposing each other. In the generalization phase, motor bias resulting from prior adaptation was observed, and it turned out to substantially depend on the property of the workspace. In Experiment 1 with a parallel workspace, preceding adaptation to the same rotation was more advantageous than adaptation to an opposing rotation. This observation was reversed in Experiment 2 with the symmetrical workspace: prior adaptation to an opposing rotation was more advantageous for the generalization than prior adaptation to the same rotation. Mechanisms possibly underlying the observed influence of the workspace configuration were discussed.

## Introduction

The phenomenon of visuomotor adaptation is abundantly investigated and well known. For instance, when in a reaching task, the visual information is spatially shifted by a prism, movements fall initially short of its target, but performance improves continuously until a more or less error-free behavior is achieved (visuomotor adaptation). Withdrawing the prism after adaptation results in the so-called aftereffect, that is in reaching errors caused by maintaining the adapted motor behavior after returning to the undistorted environment (e.g., Bedford 1993; Ooi et al. 2001; Priot et al. 2010; Redding and Wallace 1996, 2006).

Motor learning in form of visuomotor adaptation relies on updates of internal models (Wolpert et al. 1995) to counteract distorted visuomotor properties. Such model-based learning mechanisms could be either implicit or explicit (Clower and Boussaoud 2000; Hegele and Heuer 2010; Mazzoni and Krakauer 2006; Sülzenbrück and Heuer 2009). In case of implicit adaptation, minimal online corrections are—without conscious experience—automatically triggered through discrepancies between actual and desired action effects. After some repetitions, real-time error monitoring leads to an update of the internal action model, which is usually accompanied by solid aftereffects (Hinder et al. 2010; Shabbott and Sainburg 2010). Explicit adaptation is characterized by intentional control of motor action to rapidly compensate changed action dynamics. In such cases, aftereffects are usually diminished or completely absent (Hinder et al. 2008). The proportional contribution of—in most instances coexisting—implicit and explicit mechanisms of motor adaptation can vary with task features. For instance, a stepwise gradually increasing distortion allowed more complete adaptation and elicited a larger aftereffect than a sudden distortion onset. This finding suggested an increased proportion of implicit adaptation (Kagerer et al. 1997; Michel et al. 2007; Saijo and Gomi 2010).

Aftereffect is also considered as an indicator for generalization, when updated motor control is applied to other regions or other targets in the action space (e.g., Krakauer et al. 2000; Mattar and Ostry 2007) or to other body effectors (intermanual generalization; e.g., Wang and Sainburg 2003, 2005). Further, generalization could be either beneficial (transfer) or detrimental (interference; cf. Krakauer et al. 2006). Transfer occurs if the same rotation in the prior adaptation phase or a similar one is applied in the subsequent learning phase (Krakauer et al. 2000; Mattar and Ostry, 2007; Sainburg 2002; Thoroughman and Shadmehr 2000). In contrast, if the visuomotor rotation in the generalization phase was opposed, learning was interfered through the prior adaptation (Bock et al. 2001; Krakauer et al. 2005, 2006; Shadmehr and Holcomb 1999; Wang and Sainburg 2003).

**Fig. 1:**
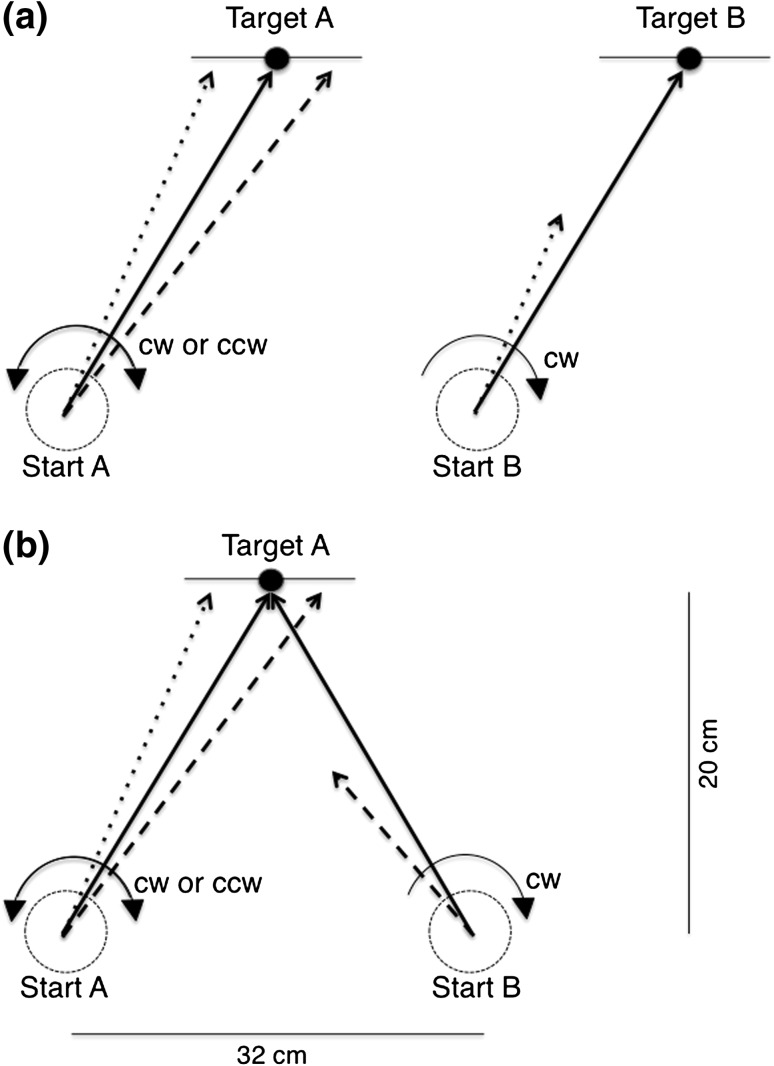
The basic procedure in the experiments. First participants aimed at a target A from a start position A on a digitizer tablet. Visual feedback on the display was clockwise (cw) or counterclockwise (ccw) rotated. After visuomotor adaptation, generalization was examined with starting from position B in the two different workspaces: the *parallel* workspace (**a**) and the *symmetrical* workspace (**b**). We hypothesized that in the *parallel* workspace, a parallel shift of movements yielded in better performance in the generalization phase (*dotted lines*), while in the *symmetrical* workspace, the mirror-inverted constellation is advantageous (*dashed lines*). The *horizontal lines* across the targets mark the reward range. The *dotted circles* (which is actually not visible to the participants) around the start positions mark the acceleration range for the aiming movement

The present study focused on the process of visuomotor generalization, more precisely, on the influence of the spatial structure of distal workspace on the generalization process. In other words, we examined whether spatial features of the workspace induce transfer or interference. In the literature, the distal workspace is casually termed “visually based extrinsic space” (e.g., Wang et al. 2010) and is referred to as the ensemble of all visual elements, which represents the action and the environment. In our case, it includes the visual representation of the start positions, the target(s), and their location to each other. Participants were seated in front of a digitizer tablet and performed aimed movements on a computer display from a start position to a target. Figure 1 shows that the left cursor movements on the screen were clockwise (cw) or counterclockwise (ccw) rotated. Successful adaptation should result in movements, which compensate for the rotation with a counter rotating correction (dotted lines for cw rotation and dashed lines for ccw rotation; Fig. 1).

After adaptation, we examined generalization in the two different workspaces: the parallel and the symmetrical workspace. In the parallel workspace (Fig. 1a), the stimulus configuration was simply shifted from the left to the right side of the monitor (start position B and target B), and accordingly, the movements were parallel shifted. The required cw adaptation at this position was concordant with the preceding cw rotation. In this case, the internal model could be simply maintained and marginally modified to fit the new effector configuration, which should facilitate the adaptation. In contrast, prior adaptation to a ccw rotation is discordant with the subsequent task requirement to adapt to an opposing—that is cw rotation—and hence make the adaptation difficult. Taken together, within this workspace, our hypothesis was in accordance with the previous studies demonstrating generalization of adaptation to adjacent movement directions, probably due to the narrow directional tuning width of the neurons involved in visuomotor adaptation (Tanaka et al. 2009).

In the symmetrical workspace (Fig. 1b), the start position was shifted from the left to the right side of the monitor, but the target remained at its position. Since the angular separation between the movement directions from the left and the right start position was as much larger (78°) than the narrow tuning width (~23°) based on the population coding model (Tanaka et al. 2009), no generalization should be observed—independent of whether a cw or a ccw rotation was applied in the adaptation phase. However, modular theories of adaptive motor control offer an alternative prediction (e.g., Haruno et al. 2001; Jacobs et al. 1991). Modular theories postulate a probabilistic estimation based on the perception of the context. In this way, the visual input selects the appropriate control module based on prior knowledge (Miall 2002). Hence, global structural similarity of the context in the adaptation and in the generalization phase is more crucial than local features like movement direction. In our case, the visual workspace formed by the start positions and the target and accordingly the movements to the target are symmetrical about the vertical axis. We suppose that this spatial regularity allows the estimator to predict a mirror-inverted scenario and further determine a transformation of the internal model into a mirror-inverted version. Hence, the preceding ccw rotation, which is opposite to the subsequent cw rotation, establishes a concordant condition and consequently results in better performance in the generalization phase compared to the discordant condition with a preceding cw rotation. Taken together, the definition of “concordance” and “discordance” is reversed compared to that in the parallel workspace. To our knowledge, the finding of a counter rotating generalization would establish a new pattern of results demonstrating the influence of the spatial workspace.

In the following experiments, we first examined the predictions within the parallel workspace (Experiment 1). Then, we examined the symmetrical workspace with unimanual (Experiment 2a) and intermanual generalization (Experiment 2b). Note, in all experiments, the advantage of the concordant condition and the disadvantage of the discordant condition are defined relatively to each other. Compared to generalization studies focusing on difference between (pre-)adaptation and post-adaptation performance (e.g., Krakauer 2009; Krakauer et al. 2006; Wang and Sainburg 2003), the core issue of the current study demands direct comparison of two different generalization conditions (concordant vs. discordant). The performance should differ between both conditions regarding the initial error or the learning rate. Consequently, the result analysis is based mainly on the performance differences between both conditions rather than on the absolute transfer or interference effect of the conditions per se.

In all experiments, aiming movements were gathered with a sliding paradigm, in which a computer cursor was flicked to a target with a rapid and short-ranged stylus movement on the graphic tablet. It is well known that movement control can be divided into an initial ballistic phase followed by a terminal phase with online correction (Medina et al. 2009). The initial vectorial movement control and the terminal correction phase obviously are based on different cognitive processes, which are playing different roles in visuomotor adaptation (Wang and Sainburg 2005). Despite great effort in analyzing the trajectory of reaching movements, a reliable separation of both components was barely achieved retrospectively in the previous studies. For the present purpose, the substantial advantage of the sliding paradigm compared to the widely used reaching movements is its focus on vectorial movement control by precluding online corrections.

## Experiment 1

Participants performed aimed sliding movements with their dominant right hand from the left start position with either a 30° cw or a 30° ccw rotation (adaptation sessions 1 and 3 in Table 1). After adaptation, they started with the same hand from the right position applying a 30° cw rotation (critical generalization sessions 2 and 4 in Table 1). In the parallel workspace (Fig. 1a), the stimulus configuration and sliding movements were simply shifted from the left to the right side, while the required movement direction remained the same. Hence, generalization condition is concordant when in the preceding session, a cw rotation is applied and discordant when a ccw rotation is applied (cf. Table 1). The concordant condition should—compared to the discordant condition—exhibit an advantage for the performance in generalization regarding the initial aiming error or/and the adaptation rate.

**Table 1:** Sequence of sessions in the experiments

Experiment	Spatial property of workspace	Session no. (block no.)	Start position	Visuomotor rotation in group 1		Visuomotor rotation in group 2	
1 Unimanual	Parallel	Baseline (block 1–2)	Left–right	0°		0°	
		1 (block 3–7)	Left	30° cw	Concordant	30° ccw	Discordant
		*2 (block 8*–*12)*	*Right*	*30° cw*		*30° cw*	
		3 (block 13–17)	Left	30° ccw	Discordant	30° cw	Concordant
		*4 (block 18*–*22)*	*Right*	*30° cw*		*30° cw*	
2a Unimanual	Symmetrical	Baseline (block 1–2)	Left–right	0°		0°	
		1 (block 3–7)	Left	30° cw	Discordant	30° ccw	Concordant
		*2 (block 8*–*12)*	*Right*	*30° cw*		*30° cw*	
		3 (block 13–17)	Left	30° ccw	Concordant	30° cw	Discordant
		*4 (block 18*–*22)*	*Right*	*30° cw*		*30° cw*	
2b Intermanual	Symmetrical	As experiment 1a					

Critical generalization sessions 2 and 4 are marked in italics

### Method

#### Apparatus and stimuli

The setup and the apparatus were the same in all experiments. The apparatus was controlled by an Apple Macintosh computer with MatLab software and the Psychophysics Toolbox (Kleiner et al. 2007). The room lights were dimmed throughout the experiment.

Participants were seated at a table. The height of the chair was adjusted individually to ensure comparable viewing and action conditions across subjects. A DIN-A3 digitizer tablet (Wacom Intuos2) resting horizontally on the table was covered by a fiberboard to block subjects’ view of their hand on the tablet. The digitizer tablet was configured in absolute position-matching mode. In this mode, each dot on the tablet was assigned to a dot on the display screen in a fixed manner.

Participants controlled the cursor movement (a small blue disk with 4 mm in diameter) on the computer display with a stylus held in their right hand. The cursor movement was displayed on a 22″ CRT color monitor (model: Iiyama Vision Master Pro514; resolution: 1,024 × 768 pixels; refresh rate: 100 Hz), which was placed upright on the table with its center at subjects’ eye level and with a distance of about 65 cm in front of the subjects.

The spatial configuration of the visual workspace is illustrated in Fig. 1. The start and target positions were marked with gray dots (5 mm in diameter) visible throughout the experiment. A gray line of 50 mm at each side of the target’s horizontal periphery served as target line marking the reward range. The distance between start positions was 32 cm, the height of the triangle 20 cm. The start positions and their respective corresponding targets constructed a right-skewed parallelogram with adjacent angles of 75° and 105°.

#### Procedure

At the beginning of each trial, the start position illuminated in yellow signalizing the valid start position, while the other start position stayed gray. Subjects had to place the cursor exactly on this start position. After staying on the start position for 500 ms, a pure tone (840 Hz) was released for 100 ms, which signaled that the trial was unlocked and the subjects had to initiate a sliding movement with their right hand as soon as possible. They were instructed to slide the cursor to a given target as precisely as possible, by accelerating the cursor with a short-ranged flicking motion of the stylus on the tablet. The flicking motion determines the proximate direction of the cursor within a radius of 2 cm around the start position. Inside this area, the cursor was controlled by stylus motion on the table for the purpose of movement initiation. Once the cursor left this area, it began to slide on a constant velocity of 17 cm/s straightly holding its direction. After the cursor hit the target line, a hit score was displayed immediately beside the final cursor position. Depending on the deviation from target middle, the hit score varied from 10 (maximum score with target middle) to 0 (minimum score 50 mm or more out of range). Individual total score gained through the experiment was multiplied by a fixed rate of 0.5 euro cent per hit point, in order to calculate the performance-based reward of each participant.

The sessions of the experiment were scheduled in Table 1. After getting acquainted with apparatus, participants performed the sliding task in 22 consecutive blocks (with 5 trials each). Session 0 with block 1 and block 2 served as baseline without visuomotor rotation. All trials of block 1 were carried out on the left start position and all trials of block 2 on the right start position.

Session 1 contained 5 blocks (block 3–7) on the left start position. A 30° rotation was introduced to alter the visual feedback during the initial acceleration throughout the session. The direction of this rotation was different between the experimental groups. For subject Group 1, it was cw and for subject Group 2 ccw. After Session 1, the starting position was shifted to the right. From there, all subjects performed the critical Session 2 (blocks 8–12) with a 30° cw rotation. Session 2 was the first crucial session gathering generalization.

Start position in Session 3 (blocks 13–17) was again at the left side. This session served to establish a ccw or cw adaptation complementary to session 1. To this end, rotations were now ccw for subject group 1 and cw for subject Group 2. In Session 4 (blocks 18–22), the critical generalization was again examined with a cw rotation. Since the rotation direction in Session 2 and Session 4 (generalization session) was always cw, it has been ensured that the crucial generalization sessions were comparable with each other—within- and between-subject groups.

After each block, a summary of hit score was provided, and the subjects could take a short break before the next block. The entire experiment lasted approximately 35 min.

#### Design and data analysis

The different sequences of the adaptation and generalization sessions between subject groups were the first independent factor of the experiment. The critical generalization sessions and the amount of blocks were within-subject factors. Thus, the experiment based on a 2 (subjects groups) × 2 (generalization sessions 2 and 4) × 5 (generalization blocks) mixed design.

As dependent variable aiming errors were gathered as angular deviations α (in degree) from the ideal trajectory. α was 0, if the actual cursor trajectory fit the ideal trajectory exactly. A positive α value indicated a clockwise deviation, and a negative α value indicated a counterclockwise deviation relative to the ideal trajectory. For statistical analysis, α-values of all experimental blocks were normalized by subtracting the baseline deviations in block 1 and block 2, respectively.

The adaptation performance in Session 1 and Session 3 was captured by analyzing the absolute aiming errors. A 5 (adaptation blocks) × 2 (groups) ANOVA was conducted for each adaptation session. The initial blocks in the generalization sessions were first compared between both groups using independent sample *t* tests. In order to examine whether the group difference in the initial generalization blocks was relying on different generalization conditions, a 2 (blocks: Block 8 and Block 18) × 2 (groups) ANOVA was conducted. The transitions from Session 1 to Session 2 and from Session 3 to Session 4 each were captured by a 2 (blocks: the last block of adaptation and the 1st block of generalization) × 2 (groups) ANOVA. Finally, the initial generalization blocks underwent a trial-by-trial analysis using 5 (trials) × 2 (groups) ANOVAs and post hoc tests.

#### Participants

Twenty naive students (10 females) took part in the experiment. Their mean age was 23.4 years (ranging from 20 to 31 years) with a standard deviation of 2.7 years. The Edinburgh Handedness Inventory yielded a mean lateralization quotient of 58.9. Participants were randomly assigned to one of the two groups.

### Results and discussion

**Fig. 2:**
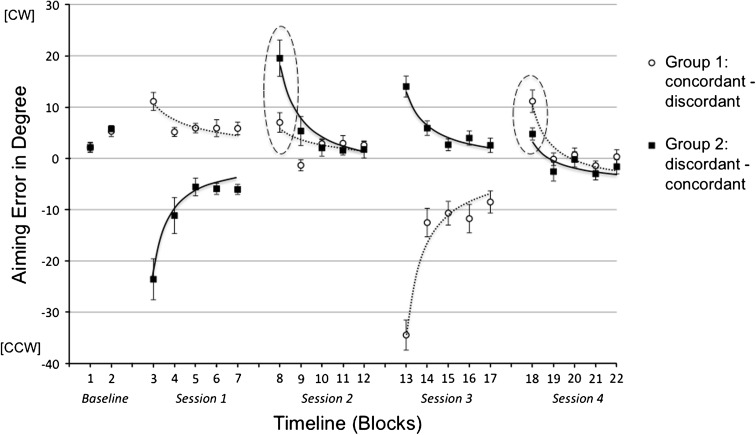
Mean aiming errors (with standard errors) in degree in the five experimental sessions of Experiment 1 broken down into 22 blocks. Every data point represents the baseline-corrected average of five consecutive trials across all subjects within the experimental groups. *Positive values* indicate deviation in cw direction, and *negative values* indicate deviation in ccw direction. *Dotted and continuous lines* represent fitted single-exponential functions (*y* = β1* × ^^^β2). Session 1 and 3 were adaptation sessions with cw or ccw rotation. Critical differences in generalization Sessions 2 and 4 are encircled with ellipsis

Results are depicted in Fig. 2. Mean aiming errors in Block 1 and Block 2 showed comparable baseline performance between the subject groups. The decline in aiming errors within each session indicated successful adaptation.

#### Adaptation sessions (Session 1 and Session 3)

The ANOVAs yielded remarkable group differences in both adaptation sessions. In Session 1, the initially large group difference diminished in the course of adaptation, which resulted in a main effect of group by trend [*F*_(1,18)_ = 3.14, *p* < .093, η^2^ = .15] and a significant group by block interaction [*F*_(4,72)_ = 8.65, *p* < .009, η^2^ = .26]. Similar results were found in Session 3 showing a main effect of group [*F*_(1,18)_ = 17.37, *p* < .001, η^2^ = .49] and a group by block interaction [*F*_(4,72)_ = 8.74, *p* < .001, η^2^ = .33] indicating a decline of the group difference over time. However, these findings had a reversed pattern showing better adaptation performance of Group 1 in Session 1 and better adaptation performance of Group 2 in Session 3, which suggested that changing rotation directions rather than subject groups were the source for the observed differences. In other words, a cw rotation was easier to be adapted than a ccw rotation, which was probably due to a greater difficulty in movement execution to counteract a ccw rotation. Significant main effect of block suggests substantial learning in both adaptation sessions (Session 1: [*F*_(4,72)_ = 19.04, *p* < .001, η^2^ = .51]; Session 3: [*F*_(4,72)_ = 49.49, *p* < .001, η^2^ = .73]). The findings in the adaptation sessions per se concern our research question only marginally, since the major focus is on the generalization sessions.

#### Generalization sessions (Session 2 and Session 4)

As marked in Fig. 2, the generalization performance of the groups differed mainly in the initial aiming errors. Aiming errors in the first block of the crucial generalization sessions were smaller in the concordant condition—that means when a cw rotation instead of a ccw rotation was adapted in the precursory session. In other words, regarding initial errors, participants were able to transfer in the concordant condition better than in the discordant condition in both Session 2 (α_concordant_ = 6.99° vs. α_discordant_ = 19.52°, [*t*_(18)_ = 3.11, *p* < .003, one-tailed]) and Session 4 (α_concordant_ = 4.75° vs. α_discordant_ = 11.13°, [*t*_(18)_ = 2.55, *p* < .01, one-tailed). This result was confirmed by a significant block (Block 8 and Block 18) by group interaction [*F*_(1,18)_ = 21.78, *p* < .001, η^2^ = .55] indicating reversed group difference caused by reversed generalization condition. Since the task requirement in the generalization session of both groups was completely identical, the observed group differences must be a product of different adaptation condition in the preceding training session. As illustrated in Fig. 2, the final adaptive state after exposure to an opposing rotation caused larger initial error in the subsequent generalization session, which resulted in significant group by block interactions in the transition from Session 1 to Session 2 [*F*_(1,18)_ = 25.24, *p* < .001, η^2^ = .58] and from Session 3 to Session 4 [*F*_(1,18)_ = 14.93, *p* < .001, η^2^ = .45].

As aforementioned, the group differences in the generalization sessions were located mainly in the first block, but not in the later adaptation blocks, which indicates different initial motor bias as the primary source of the group difference rather than generalization, which affects particularly the adaptation rate (Krakauer et al. 2006). Thus, more stronger group differences should be observed in the first few trials. Additionally, with respect to the introduced rotation of 30°, angular errors in both Block 8 and Block 18 were on a remarkably low level for both groups, which could be caused by an extensive transfer effect or by a strong training effect within a block. It makes a breakdown of the blocks into single trials meaningful. Accordingly, single-trial analysis was conducted for Block 8 and Block 18.

**Fig. 3:**
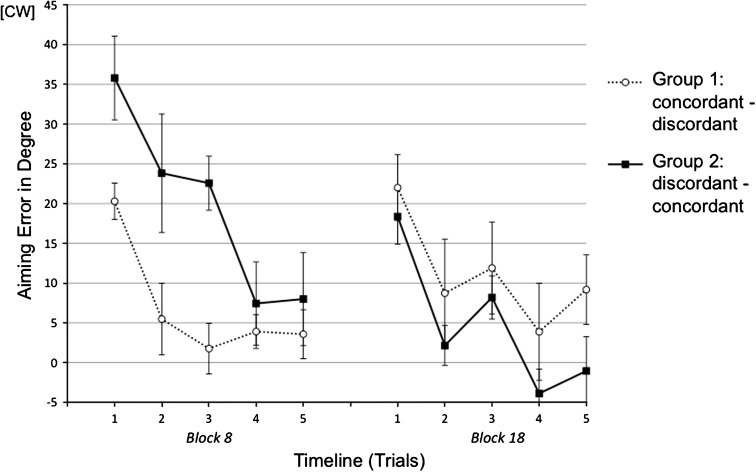
Mean aiming errors (with standard errors) of the initial block of Session 2 (Block 8) and the initial block of Session 4 (Block 18) in single-trial analysis of Experiment 1. Both blocks were broken down into 5 trials each. Every data point represents the average of all subjects within an experimental group

As illustrated in Fig. 3, Block 8 showed initially large errors followed by a rapid decline. A 5 (trials) × 2 (groups) mixed ANOVA yielded significant main effect of group [*F*_(1,18)_ = 9.68, *p* < .006, η^2^ = .35], which was in line with the group differences reported in the previous section. More importantly, post hoc tests with Bonferroni correction (*p* = .010) yielded large group differences in the first [*t*_(18)_ = 2.70, *p* < .008, one-tailed] and the third trial [*t*_(18)_ = 4.46, *p* < .001, one-tailed], and a trend in the second trial [*t*_(18)_ = 2.11, *p* < .025, one-tailed]. For Block 18, a 5 (trials) × 2 (groups) mixed ANOVA yielded significant main effect of group [*F*_(1,18)_ = 6.49, *p* < .02, η^2^ = .27]. Even though no significant group difference was found in post hoc comparisons of single trials, a trial (the first trial in Block 8 and the first trial in Block 18) by group interaction by trend was found [*F*_(1,18)_ = 4.27, *p* < .054, η^2^ = .19] indicating reversed group difference caused by reversed generalization condition.

## Experiment 2a and 2b

Since Experiment 1 brought evidence for the advantage of preceding adaptation to the same rotation for the subsequent generalization in a parallel workspace, which is consistent with the previous findings in the literature, Experiment 2 aimed to demonstrate the reversed finding. In the symmetrical workspace (Fig. 1b), the relationship between workspace and visuomotor rotation is concordant when in the preceding session a ccw rotation was introduced, and discordant when a cw rotation was applied (Table 1). In accordance with our hypotheses outlined in the Introduction, we expected better performance in the generalization session in the concordant condition compared to the discordant condition. And this effect should be mainly pronounced in the initial aiming errors as shown in Experiment 1. Experiment 2a and 2b focused respectively on intramanual and intermanual generalization.

### Method

#### Apparatus and stimuli

The basic design remained the same as in Experiment 1. The major difference between experiments was the spatial feature of the visual workspace. In Experiment 2a and 2b, two start positions and a target shaped an isosceles triangle with base angles of 51° (Fig. 1b).

#### Procedure of Experiment 2a

The sessions of the experiment are scheduled in Table 1. In Session 1, participants performed aimed sliding movements from the left start position with either a 30° cw (Group 1) or a 30° ccw (Group 2) rotation. After adaptation, they started from the right start position with a 30° cw rotation in Session 2. Given the symmetrical workspace, the generalization condition was disconcordant for Group 1 and concordant for Group 2.

The rotation in the second adaptation session (Session 3) had the same magnitude but reversed direction in both subject groups, which means ccw for Group 1 and cw for Group 2. Since the rotation in the subsequent generalization session (Session 4) remained 30° cw, the generalization condition was reversed as well, which means concordant for Group 1 and discordant for Group 2. All movements were again performed with the dominant right hand. Hence, the transition from the adaptation sessions to the proximate generalization constituted an intramanual transfer scenario.

#### Procedure of Experiment 2b

Experiment 2b was based on the same procedure as Experiment 2a with only one change: in Experiment 2b, each start position was assigned to the laterally corresponding hand. Consequently, adaptation Session 1 and Session 3 were performed with the left hand, whereas generalization Session 2 and Session 4 were performed with the dominant right hand. Hence, the transition from the adaptation sessions to the proximate generalization constituted an intermanual transfer scenario.

#### Design and data analysis

Experiment 2a and 2b had the same 2 (subjects groups) × 2 (generalization sessions 2 and 4) × 5 (generalization blocks) mixed design as Experiment 1. And again, angular deviation α (in degree) from the ideal trajectory was registered as dependent variable, which was normalized by subtracting the baseline deviation in Block 1 and Block 2, respectively. The normalized aiming errors underwent the same statistical analyzes as described in Experiment 1.

#### Participants

Eighteen right-handed students (15 females) from RWTH Aachen University took part in Experiment 2a and twenty other right-handed students (13 females) in Experiment 2b. Participation in the experiments was reimbursed with 5 € plus a performance-based reward of max. 5 €. In Experiment 2a, the mean age of participants was 22.5 years (ranging from 19 to 28 years) with a standard deviation of 2.2 years; in Experiment 2b, it was 22.6 years (ranging from 19 to 29 years) with a standard deviation of 2.9 years. Handedness was ensured with the Edinburgh Handedness Inventory (mean lateralization quotients of 76.8 in Experiment 2a and 70.0 in Experiment 2b; Oldfield 1971). In both experiments, participants were randomly assigned to one of the two groups.

### Results Experiment 2a

**Fig. 4:**
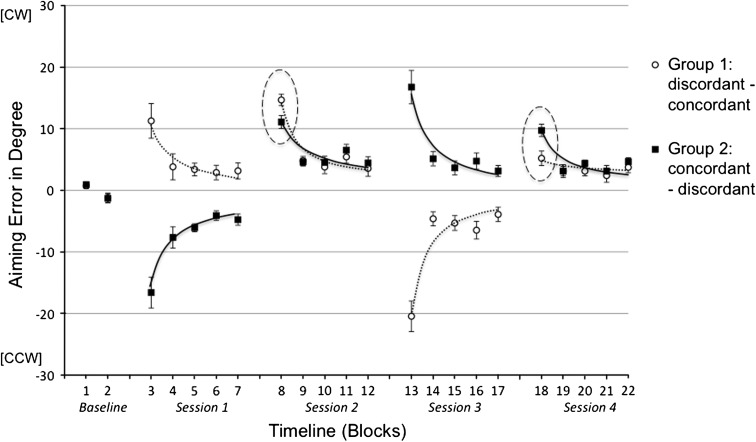
Mean aiming errors (with standard errors) in degree in the five experimental sessions of Experiment 2a broken down into 22 blocks. Every data point represents the baseline-corrected average of five consecutive trials across all subjects within the experimental groups. *Positive values* indicate deviation in cw direction, and *negative values* indicate deviation in ccw direction. *Dotted and continuous lines* represent fitted single-exponential functions (*y* = β1* × ^^^β2). Session 1 and 3 were adaptation sessions with cw or ccw rotation. Critical differences in generalization Session 2 and 4 are encircled with ellipsis

In the baseline measure (Block 1 and Block 2 in Fig. 4), marginal inherent aiming bias was observed, which was comparable between both subject groups. The time courses of adaptation in all experimental sessions were quantified by fitting single-exponential functions to the group mean data.

#### Adaptation sessions (Session 1 and Session 3)

Analysis of the absolute aiming errors in Session 1 and Session 3 using 5 (adaptation blocks) × 2 (groups) ANOVAs yielded neither main effect of group (*p* > .23) nor group by block interaction (*p* > .36). Significant main effect of block suggests substantial learning in both adaptation sessions (Session 1: [*F*_(4,64)_ = 52.42, *p* < .001, η^2^ = .77]; Session 3: [*F*_(4,64)_ = 42.89, *p* < .001, η^2^ = .73]). For the present research question, the more important findings regarding the generalization performance in dependence of final adaptive states are reported in the following section.

#### Generalization sessions (Session 2 and Session 4)

The main results were observed in the critical generalization sessions 2 and 4, which showed group difference regarding the initial aiming error. In accordance with our hypotheses, initial performance (Block 8) in Session 2 was better after ccw rotation in Session 1, which established a concordant condition for the generalization (α_concordant_ = 11.10 vs. α_discordant_ = 14.68°, [*t*_(16)_ = 1.93, *p* < .036, one-tailed]). The same pattern of finding was observed in Session 4 with a tendentially smaller initial aiming error (Block 18) in the concordant condition (α_concordant_ = 5.20° vs. α_discordant_ = 9.73°, [*t*_(16)_ = 1.62, *p* < .063, one-tailed]). This result was confirmed by a significant block (Block 8 and Block 18) by group interaction [*F*_(1,16)_ = 13.32, *p* < .002, η^2^ = .45] indicating a reversed group difference caused by a reversed generalization condition. We further evaluated these group differences in background of different adaptation conditions in the preceding training session, which according to the experimental variation must be the only cause of this between-subject effect. As marked in Fig. 4, significant group effects were observed in the transition from Session 1 to Session 2 [*F*_(1,16)_ = 34.28, *p* < .001, η^2^ = .68] and from Session 3 to Session 4 [*F*_(1,16)_ = 11.98, *p* < .003, η^2^ = .43]. In both cases, no group by block interaction was found, which means—in contrast to Experiment 1—the final adaptive state after exposure to an opposing rotation did not cause a larger but a smaller initial error in the subsequent generalization session.

**Fig. 5:**
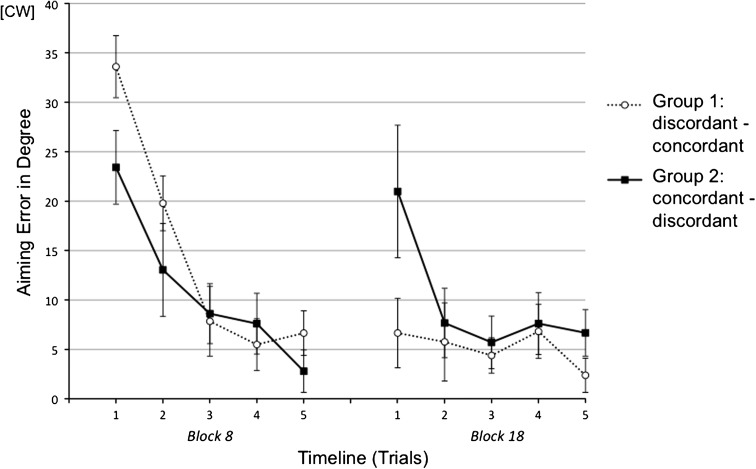
Mean aiming errors (with standard errors) of the initial block of Session 2 (Block 8) and the initial block of Session 4 (Block 18) in single-trial analysis of Experiment 2a. Both blocks were broken down into 5 trials each. Every data point represents the average of all subjects within an experimental group

As Experiment 1, the group differences in the generalization sessions were located mainly in the first block. Since the initial motor bias should be more pronounced in the first few movements, we expected stronger group differences by conducting single-trial analysis for Block 8 and Block 18. Indeed, single-trial analysis shown in Fig. 5 yielded significant group differences in the first trial of Block 8 (α_concordant_ = 23.42° vs. α_discordant_ = 33.61°, [*t*_(16)_ = 2.09, *p* < .027, one-tailed]) and in the first trial of Block 18 (α_concordant_ = 6.66° vs. α_discordant_ = 20.98°, [*t*_(16)_ = 1.89, *p* < .039, one-tailed]). Accordingly, a 2-trial (the initial trial in Block 8 and the initial trial in Block 18) × group ANOVA showed a significant trial by group interaction [*F*_(1,16)_ = 9.95, *p* < .006, η^2^ = .39] indicating reversed group difference caused by reversed generalization condition.

### Results Experiment 2b

**Fig. 6:**
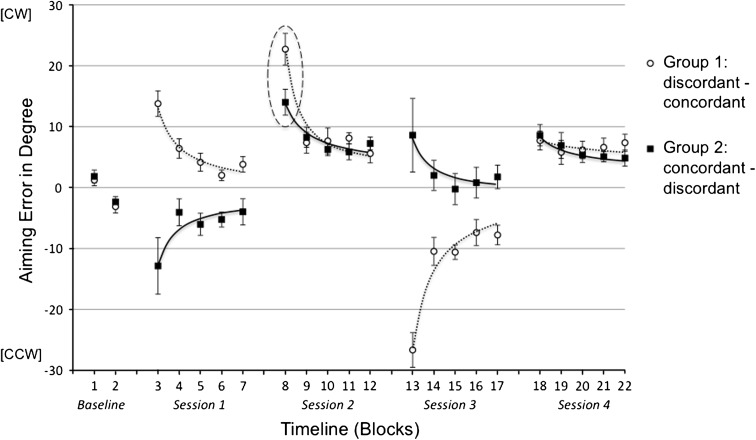
Mean aiming errors (with standard errors) in degree in the five experimental sessions of Experiment 2b broken down into 22 blocks. Every data point represents the baseline-corrected average of five consecutive trials across all subjects within the experimental groups. *Positive values* indicate deviation in cw direction, and *negative values* indicate deviation in ccw direction. *Dotted and continuous lines* represent fitted single-exponential functions (*y* = β1* × ^^^β2). Session 1 and 3 were adaptation sessions with cw or ccw rotation. Critical differences in generalization Session 2 and 4 are encircled with ellipsis

Results of Experiment 2b are depicted in Fig. 6. Both groups showed again comparable performance in the baseline measure.

#### Adaptation sessions (Session 1 and Session 3)

Absolute aiming errors in Session 1 and Session 3 were analyzed using 5 (adaptation blocks) × 2 (groups) ANOVAs. In Session 1, no significant group effect (*p* > .29) or group by block interaction (*p* > .81) was found. In contrast, a significant group main effect [*F*_(1,18)_ = 5.26, *p* < .034, η^2^ = .23] was observed in Session 3 showing better adaptation performance of Group 2 (cw rotation) compared to Group 1 (ccw rotation). Significant main effect of block suggests progressive reduction of aiming error in both adaptation sessions (Session 1: [*F*_(4,72)_ = 21.26, *p* < .001, η^2^ = .54]; Session 3: [*F*_(4,72)_ = 29.92, *p* < .001, η^2^ = .62]). In the following section, we focused on the more relevant issue concerning the group differences in the generalization sessions with respect to the respective preceding final adaptive states.

#### Generalization sessions (Session 2 and Session 4)

**Fig. 7:**
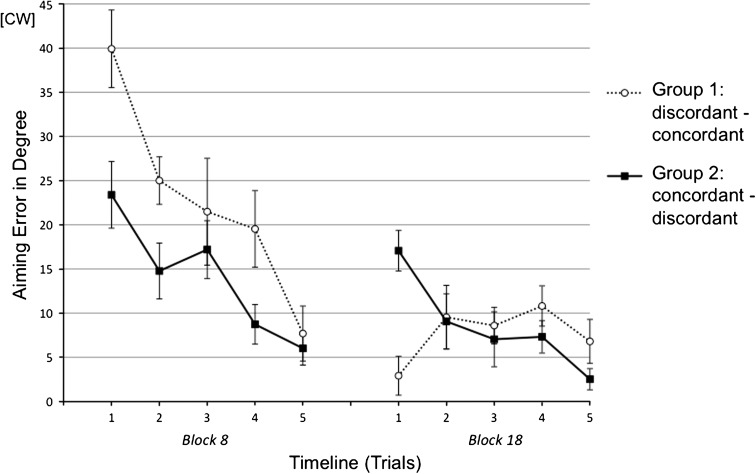
Mean aiming errors (with standard errors) of the initial block of Session 2 (block 8) and the initial block of Session 4 (block 18) in single-trial analysis of Experiment 2b. Both blocks were broken down into 5 trials each. Every data point represents the average of all subjects within an experimental group

Comparison of experimental groups regarding the aiming performance in the critical generalization sessions yielded consistent findings with those of Experiment 2a. Smaller aiming error in the first block (Block 8) of Session 2 was observed, when the preceding adaptation was ccw, which means the condition was concordant (α_concordant_ = 14.01° vs. α_discordant_ = 22.72°, [*t*_(18)_ = 2.59, *p* < .009, one-tailed]). Further inspection of this group difference in background of the final adaptive state of Session 1 via a 2 blocks (final block in session 1 and first block in Session 2) × 2 groups ANOVA showed significant main effect of group [*F*_(1,18)_ = 16.06, *p* < .001, η^2^ = .47] and no block by group interaction (*p* > .83). This result indicated the final adaptive state after exposure to an opposing rotation did not cause a larger but a smaller initial error in the subsequent generalization session. Even though no significant group difference in the initial block (Block 18) in Session 4 was found, single-trial analysis (Fig. 7) corroborated the advantage of opposing adaptation for the subsequent generalization by showing large group differences in the first trial of both Block 8 (α_concordant_ = 23.38° vs. α_discordant_ = 39.91°, [*t*_(18)_ = 2.86, *p* < .005, one-tailed]) and Block 18 (α_concordant_ = 2.90° vs. α_discordant_ = 17.06°, [*t*_(18)_ = 2.59, *p* < .001, one-tailed]). Additionally, a 2-trial (the initial trial in Block 8 and the initial trial in Block 18) × group ANOVA showed a significant trial by group interaction [*F*_(1,18)_ = 32.26, *p* < .001, η^2^ = .76] indicating reversed group difference caused by reversed generalization condition.

### Discussion

In accordance with our hypotheses, both Experiment 2a and 2b demonstrated group differences in favor of opposing rotation in a symmetrical workspace. Furthermore, the advantage of the opposing rotation was observed mainly in the first blocks of both generalization sessions. Hence, the effect relies primarily on a motor bias rather than generalization of learning process, which should pronounce mainly in adaptation rate. We assume that the motor control is guided by the a priori hypotheses about the property of the action environment, whereby the spatial particularity of the workspace was taken into account.

## General discussion

The present study attempts to get a better understanding of visuomotor adaptation and generalization by taking the spatial property of the distal workspace into account. The findings of the previous studies suggest that transfer occurs when the rotation is maintained for other regions, other targets in action space, or other body effectors (Krakauer et al. 2000; Mattar and Ostry 2007; Sainburg 2002; Thoroughman and Shadmehr 2000). More importantly, these studies have also shown that visuomotor adaptations have narrow generalization functions regarding movement directions, that is, learning in one movement direction only affects subsequent movements into adjacent directions. However, the present study demonstrated that the way prior adaptation affects the subsequent action depends on the spatial property of the workspace.

The findings coincide with those reported by Wang et al. (2010). In that study, subjects performed bilateral movements to targets either in the same or opposing directions, while the visuomotor rotations altering the feedback of the movements were either the same or opposing to each other as well. The results indicated minimal bilateral interference when both target directions and visual rotation directions were parallel or symmetrical (corresponds to the concordant conditions in Experiment 1 and Experiment 2 in the current study) between the arms. In spite of differences in the tasks, both studies are in agreement with the idea that visual information processing and global structural similarity of the visual context play a major role in optimal motor learning. In the present study, the influence of the visual action context was demonstrated regarding two aspects.

Firstly, we could show reversed effects of prior adaptation across the experiments (Experiment 1 vs. Experiment 2). In the parallel workspace (Experiment 1), adaptation to the same (cw) rotation was beneficial and enabled better performance in the generalization phase than prior adaptation to an opposing (ccw) rotation. In a symmetrical workspace (Experiment 2a and 2b), prior adaptation to an opposing (ccw) rotation was more advantageous for the performance in the generalization phase. Consequently, the concordance or discordance between prior adaptation and subsequent generalization has turned out to be a function of the workspace, even though the differences between concordant and discordant conditions were limited to the initial performance. It is also essential to stress that the difference between the concordant and discordant conditions is not necessarily a result of a better transfer in the concordant setting. Although the single-trial analyses in the concordant generalization condition showed some positive transfer reducing the initial aiming error caused by the visuomotor rotation, the group effects seemed to be driven primarily by interference in the discordant condition. To estimate the transfer and interference and their partial contributions to the group difference, post-adaptation performance has to be measured without visuomotor rotation. In this way, aftereffects as the driving force for either transfer or interference can be quantified precisely. Hence, it remains interesting and meaningful to extent the present findings in future work.

Secondly, in Experiment 2a and 2b, prior adaptation affected subsequent action, although the angular separation between the required movement directions in the adaptation and the generalization phase was extremely large. As we have reasoned in the Introduction, this finding contradicts the previous findings indicating that generalization was confined to adjacent movement directions (e.g., Krakauer et al. 2000; Sainburg 2002; Thoroughman and Shadmehr 2000; Mattar and Ostry 2007). It remains open how the configuration of the task environment intervened in the generalization process and caused the motor bias. We discuss three different approaches, which do not have to be mutually exclusive.

The first approach suggests a mechanism involving primarily the predictive process in motor control selection. The internal model simulates the forward action flow (Miall and Wolpert 1996; Shadmehr et al. 2010) by taking environmental parameters into account such as gravitation, frictional force, or visuomotor rotation. Since coexistence of different adaptation states has been shown to be possible (e.g., Bock et al. 2005; Lee and Schweighofer 2009), it has to be decided which model should be applied. Maybe, if the situation is ambiguous, the motor system deals with the situation in a “conservative” way, by using the inherent model. A priori selection could be made using a prior probabilistic estimation based on perception of the context (e.g., Haruno et al. 2001; Jacobs et al. 1991; Miall 2002). However, if the context is ambiguous, the selection has to be postponed until the action environment is explored. In the study of Wang and Sainburg (2003) focusing on interlimb generalization of visuomotor rotation, opposite arm training was generalized to the subsequent movements of the other arm. However, it did not effect the first movements made during subsequent performance. That means the effects of opposite arm training did not occur until finishing the very first movement. The authors argued that the first trials of generalization were used to probe current movement conditions to determine whether to use opposite arm derived information.

Obviously, participants in the present study had no such problems due to situational uncertainty. Prior experience with the workspace and accordingly the visuomotor rotation seems to form unambiguous expectation for the upcoming situation. The motor control was therefore guided by an a priori hypotheses based on the perception of the workspace. Consequently, the effects in the present study were mainly pronounced in initial directional biases. It supports our assumption that a representation of the action space, which includes the visuomotor rotation as an integrative spatial aspect, is fundamental for action control.

The second approach suggests a mechanism involving primarily the perceptual processes. The processes of adaptation and generalization in the symmetric workspace might be modulated by the so-called perceptive realignment (Redding and Wallace 1996, 2006). In the symmetric workspace, errors in perceiving the target distance would cause the same aiming error as in case of visuomotor rotations: Perceiving a target as closer would lead to a negative α error like a rotation inwards, while perceiving a target as further away would cause a positive α error like a rotation outwards. Conversely, successful adaptation to visuomotor rotations could be based on a perceptual realignment by generating a virtual target with appropriate height for the motor planning. In a current study in our laboratory, we are investigating this issue regarding the reciprocal influences between motor adaptation and visual perception of the space.

The third approach provides an explanation based on model-free learning process. Repetition of the newly adapted movement induces directional biases toward the repeated movement (Verstynen and Sabes 2011), which is termed “use-dependent learning”. It can lead to persistent movement changes and account for generalization as well (Diedrichsen et al. 2010). Since no explicit model of the perturbation is necessary, it is considered as model free and usually “hidden” behind the adaptation (Huang et al. 2011). In this case, instead of the workspace, the hand movement direction becomes more critical. In Experiment 1, directional bias in the generalization phase was consistent with the repeated hand direction in the adaptation phase. It seems to perfectly fit the prediction based on use-dependent learning. Since the parallelism applied equally to the workspace and the hand direction without further experimental distinction, one may argue that solely the hand direction was responsible for the generalization effect. Also in Experiment 2b, the symmetry applied equally to the distal workspace and the proximal hand movement direction. The movements were carried out at the left and right start position with the left and the right hand, respectively. Hence, the postural configuration and the muscle units recruited for the movements with the left and right hand were symmetrical. Consequently, the symmetrical hand configuration could be considered to explain the observed symmetrical motor bias completely. However, in Experiment 2a, there was no postural symmetry, since the movements at both start positions were executed with the same (right) hand. The joint configuration and the involved muscle units were very different. In this case, it is difficult to explain the symmetrical movement bias solely with hand movement symmetry. Hence, we believe that the spatial property of the workspace but not the movement direction configuration was the decisive factor for the observed motor bias. Nevertheless, it remains interesting to examine all three alternatives in a future study.

## Conclusion

Generalization of visuomotor adaptation is substantially influenced by the prior experience with the action and the concordance with the subsequent situation. However, the concordance must be defined with respect to the particular feature of distal action space, since the present study has shown that motor bias in the generalization phase could be reversed by varying the spatial structure of distal action space. We therefore suggested a systemic approach for sensorimotor transformations by regarding them as an integrative part of the workspace.
